# Lactic acid in the vaginal milieu modulates the *Candida*-host interaction

**DOI:** 10.1080/21505594.2025.2451165

**Published:** 2025-01-22

**Authors:** Diletta Rosati, Marisa Valentine, Mariolina Bruno, Arnab Pradhan, Axel Dietschmann, Martin Jaeger, Ian Leaves, Frank L. van de Veerdonk, Leo A.B. Joosten, Sumita Roy, Mark H. T. Stappers, Neil A.R. Gow, Bernhard Hube, Alistair J.P. Brown, Mark S. Gresnigt, Mihai G. Netea

**Affiliations:** a Department of Internal Medicine and Radboud Center for Infectious Diseases, Radboud University Medical Center, Nijmegen, T he Netherlands; b Department of Microbial Pathogenicity Mechanisms, Leibniz Institute for Natural Product Research and Infection Biology - Hans-Knöll-Institute, Jena, Germany; c Medical Research Council Centre for Medical Mycology, University of Exeter, Exeter, UK; d Junior Research Group Adaptive Pathogenicity Strategies, Leibniz Institute for Natural Product Research and Infection Biology - Hans-Knöll-Institute, Jena, Germany; e Department of Medical Genetics, Iuliu Hatieganu University of Medicine and Pharmacy, Cluj-Napoca, Romania; f Institute of Microbiology, Friedrich-Schiller-University, Jena, Germany; g Department of Immunology and Metabolism, Life and Medical Sciences Institute (LIMES), University of Bonn, Bonn, Germany

**Keywords:** Vulvovaginal candidiasis, *candida* albicans, host response, lactic acid, vaginal simulative medium

## Abstract

Vulvovaginal candidiasis (VVC) is one of the most common infections caused by *Candida albicans*. VVC is characterized by an inadequate hyperinflammatory response and clinical symptoms associated with *Candida* colonization of the vaginal mucosa. Compared to other host niches in which *C. albicans* can cause infection, the vaginal environment is extremely rich in lactic acid that is produced by the vaginal microbiota. We examined how lactic acid abundance in the vaginal niche impacts the interaction between *C. albicans* and the human immune system using an *in vitro* culture in vaginal simulative medium (VSM). The presence of lactic acid in VSM (VSM+LA) increased *C. albicans* proliferation, hyphal length, and its ability to cause damage during subsequent infection of vaginal epithelial cells. The cell wall of *C. albicans* cells grown in VSM+LA displayed a robust mannan fibrillar structure, β-glucan exposure, and low chitin content. These cell wall changes were associated with altered immune responses and an increased ability of the fungus to induce trained immunity. Neutrophils were compromised in clearing *C. albicans* grown in VSM+LA conditions, despite mounting stronger oxidative responses. Collectively, we found that fungal adaptation to lactic acid in a vaginal simulative context increases its immunogenicity favouring a pro-inflammatory state. This potentially contributes to the immune response dysregulation and neutrophil recruitment observed during recurrent VVC.

## Introduction

Although *C. albicans* is generally a harmless colonizer of mucosal surfaces, under certain circumstances it can infect the vaginal mucosa and cause vulvovaginal candidiasis (VVC) [1], having a strong impact on quality of life and mental wellbeing [2,3]. VVC affects around 75–80% of women worldwide at least once in their lifetime, while up to 9% of women, representing more than 100 million, report four or more episodes per year – a condition termed recurrent VVC (RVVC) [4,5]. Several factors, such as excessive antibiotic usage, diabetes mellitus, hormone therapy, and pregnancy, increase the likelihood of VVC [6]. However, in most women recurrent infections are not associated with these risk factors and therefore a comprehensive understanding of VVC/RVVC pathophysiology is still lacking.

The *C. albicans* cell wall is a two-layered structure formed by chitin, glucans, and mannosylated proteins, which can all act as pathogen-associated molecular patterns (PAMPs) [7] that are recognized by pattern recognition receptors (PRRs) expressed by innate immune cells. This engagement triggers phagocytosis and fungal clearance *via* oxidative and non-oxidative antimicrobial mechanisms [8]. In addition, immune cells release pro-inflammatory cytokines that further activate the innate and adaptive immune responses that ultimately confer long-term memory against reinfection [8,9]. In a healthy host, these combined mechanisms induce protective immunity, resulting in pathogen clearance and resolution of the infection [8]. However, during RVVC this response has been suggested to be dysfunctional. Hyperinflammation during RVVC results from a strong activation of inflammatory responses by vaginal epithelial cells and the NLRP3 inflammasome in macrophages [10–12]. These responses drive the recruitment of neutrophils that are unable to clear infection at the vaginal epithelium, which ultimately leads to immunopathology characterized by host tissue damage [10,13–16].

Complementing classical adaptive immunity, extensive research has shown that myeloid cells pre-exposed to either *C. albicans* or exogenous stimuli display increased responsiveness to a secondary non-specific insult or PRRs *via* epigenetic and metabolic reprogramming [17]. This mechanism of innate immune memory is called “trained immunity” and has been shown to boost host defences, thus exerting additional beneficial effects [18]. Further, epithelial stem cells have been shown to display features of innate immune memory, which confer rapid protection at the niche barrier against pathogens and maintain tissue homoeostasis [18,19]. Trained immunity is therefore likely to play a role during vaginal *C. albicans* infection, although this has not yet been investigated.

Intrinsically different from other body niches commonly colonized by *Candida*, the vaginal microenvironment is mostly dominated by *Lactobacillus* species [20], which acidify the pH of the vaginal lumen [21]. Furthermore, lactic acid produced by the microbiota is highly abundant in the vaginal niche [22]. Perturbation of the vaginal microbiota, for example by antibiotic treatment, is associated with the overgrowth of *C. albicans* and symptomatic disease in RVVC patients [23], which may associate with the capacity of lactobacilli to antagonize the growth of *C. albicans* [24–27]. Yet, in contrast to bacterial vaginosis, the pH and abundance of lactic acid in the vagina are not strongly affected during RVVC [28–30].

Weak carboxylic acids, like lactic acid from the vaginal microenvironment, have been shown to impact *C. albicans* cell wall architecture, expression of virulence factors, resistance to antifungals, and recognition by host immune cells [31–33]. However, most of these studies made use of “standard” *in vitro* culture media such as yeast extract-peptone-dextrose (YPD), which only to a limited extent can be used to draw conclusions about the influence of lactic acid in the *in vivo* vaginal environment [33,34]. The importance of using conditions mirroring the vaginal environment, such as a vaginal simulative medium (VSM), has been addressed by Moosa and co-workers who reported that *C. albicans* susceptibility to fluconazole treatment differed between VSM and yeast nitrogen base (YNB) medium [35]. Therefore, to better characterize the effect of the specific vaginal microenvironment on the interaction between *C. albicans* and the host in the context of VVC, we investigated the impact on the *C. albicans*-host interaction of exposure to lactic acid under conditions resembling the vaginal microenvironment *in vitro*. To this end, *C. albicans* was grown in VSM containing or lacking lactic acid. Using this experimental approach, we assessed how growth in an environment with abundant lactic acid impacts *C. albicans* virulence traits, cell wall architecture, and its interaction with immune cells. By evaluating different host cell types, including vaginal epithelial cells, peripheral blood mononuclear cells (PBMCs), macrophages, and neutrophils we systematically dissected how the presence of lactic acid in the vagina may shape RVVC immunopathogenesis. We found that growth in lactic acid in VSM affects *C. albicans* properties and pathogen-host interactions, which could contribute to a general pro-inflammatory state in the host.

## Materials and methods

### Medium and stimuli preparation

To mimic the vaginal luminal environment, vaginal simulative medium (VSM) was prepared according to Roparts *et al.*, [36] to contain 58 µM NaCl, 18 mM KOH, 0.013% (v/v) glycerol, 2 mM Ca(OH)2, 6.7 mM urea, 33 mM glucose, 0.67% (w/v) yeast nitrogen base (YNB), 21.73 mM lactic acid, and 17 mM acetic acid. To specifically investigate the influence of lactic acid in the vaginal milieu, VSM was prepared in the presence (VSM+LA) or absence of lactic acid (VSM-LA). DL-lactic acid (Sigma, catalogue number 69785) was used at a concentration of 21.73 mM, which falls within the physiological range commonly found in the vaginal environment of women [37]. Both media were buffered using HCl to a vaginal pH of 4.5, typical of women of reproductive age [38] and then filter-sterilized using a 0.22 µm filter. Where indicated, VSM was buffered to a pH of 6 using NaOH before being filter-sterilized.

*Candida albicans* SC5314 [39] was streaked from a glycerol stock and maintained on yeast extract-peptone-dextrose (YPD) agar plates. A single colony from the agar plate was inoculated in liquid VSM or YPD medium (for control experiments) and incubated overnight at 30°C while shaking at 140 rpm. Fungal cells were harvested by centrifugation at 2000 rpm for 8 minutes (min), washed three times with phosphate buffered saline (PBS, pH 7.4), and counted using a Neubauer chamber (Merck). Live or killed yeast cells were used in the study. To prepare killed *C. albicans*, washed cells were either heat-killed (HK) for 30 min in a water bath at 95°C or incubated with 50 mM Thimerosal (Sigma-Aldrich) overnight at room temperature (RT) in the dark. The cells were then washed three times with PBS, counted using a Neubauer chamber, and adjusted to a final concentration of 1 × 10^8^ fungal cells/mL in PBS. The killing efficiency was proved by the absence of growth on Sabouraud agar plates.

*Escherichia coli* ATCC35218 was streaked from a glycerol stock and inoculated in Brain-Heart Infused (BHI; Becton Dickinson) medium for a maximum of 16 h at 37°C while shaking at 140 rpm. Bacterial cells were then harvested by centrifugation at 3000 rpm for 10 min at 4°C, washed three times in PBS, and resuspended in 10 mL of PBS. Bacterial cells were serially diluted: the 6 lowest dilutions were plated onto 5% Sheep Blood Agar plates (Becton Dickinson) using the spot plating technique, followed by overnight incubation at 37°C. The remaining bacterial cells were heat-killed at 95°C for 30 min in a water bath. After overnight incubation, isolated colonies per spot were counted and the number of cells from the heat-killed stock was brought to a final concentration of 1 × 10^9^ bacterial cell/mL, then stored at −20°C until further use.

### *C. albicans* growth curves

To test growth of *C. albicans* SC5314 cultured in VSM with or without lactic acid, washed overnight cultures were resuspended in Roswell Park Memorial Institute 1640 (RPMI, supplemented with glutamine; Gibco) medium and 4 × 10^5^ yeast cells/well were incubated in flat-bottom 96-well plates. To investigate fungal oxidative stress resistance, H_2_0_2_ (10 mM) or H_2_0 (as control) was added to 4 × 10^5^ *C. albicans* cells/well in flat-bottom 96-well plates. Fungal growth was monitored by measuring the absorbance at 600 nm in a Tecan microplate reader every 30 min for 24 h at 37°C, before each measurement, the plate was shaken for 15 seconds (s) at 140 rpm. Significance was analysed using GraphPad Prism v.8.0.2 by using a paired t-test.

### Hyphal length

1 × 10^5^ yeast cells/mL from overnight cultures in VSM±LA were added to μ-Slide 8 Well (IBIDI) and incubated for 3 h at 37°C in RPMI. Following incubation, the supernatant was removed, and cells were fixed with 4% Histofix for 15 min at 37°C. Cells were washed, resuspended in PBS, and stored at 4°C until imaging. Images were acquired using a Zeiss Observer microscope (Carl Zeiss) at 10× magnification. Hyphal length was quantified using the line tool in FIJI software [40].

### Green fluorescent protein (GFP) reporter strains

Green fluorescent protein (GFP) reporter strains were used as previously described [41]. Specifically, *C. albicans* CTA1p-GFP, which expresses GFP under the control of the CTA1 promoter, yeast cells carrying *pGFP* (promotor-less GFP gene, negative control), and *ACT1p-GFP* (under the control of the actin gene promoter, positive control) were grown overnight in VSM with or without lactic acid. After three washes in PBS, the GFP reporter strains were brought to a final concentration of 8 × 10^6^ yeast cells/mL and exposed to 2 mM H_2_O_2_ for 2 h at 37°C. Following incubation, cells were centrifuged and fixed with 4% Histofix for 15 min at 37°C. Finally, samples were washed once, resuspended in PBS, and the fluorescence signal was quantified using a BD FACSVerse CellAnalyser and analysed using FlowJo software (V10).

### Culture, maintenance, and infection of vaginal epithelial cells

Human A-431 vaginal epithelial cells (VECs; ACC 91) were cultured in RPMI supplemented with 10% heat-inactivated foetal bovine serum (FBS; Bio&Sell) at 37°C and 5% CO_2_. The cell line was authenticated *via* commercial STR profiling (Eurofins Genomic) and checked for mycoplasma contaminations using a PCR mycoplasma test kit (PromoKine) according to the manufacturer’s instructions.

For infection experiments, VECs were seeded at densities of 2 × 10^4^ cells/well and 1 × 10^5^ cells/well in flat-bottom 96-well and 24-well plates, respectively. After 2 days, confluent epithelial cells in 96-well plates were infected with 50 µl of 4 × 10^5^ *C. albicans* cells/mL [multiplicity of infection (MOI) 1; final volume of 200 µL with RPMI] and incubated for 24 h at 37°C and 5% CO_2_. After 24 h, plates were centrifuged at 200 *g* for 10 min and supernatants were collected for further assessment or stored at −20°C until cytokine evaluation.

### Cytotoxicity quantification

Following 24 h of infection, VEC damage was quantified by measuring activity of the cytoplasmatic enzyme lactate dehydrogenase (LDH) in collected supernatants using a cytotoxicity detection kit (Roche) according to the manufacturer’s instructions.

### Determination of *C. albicans* colony forming units

Twenty-four hours after infection, infected VECs in 96-well plates were treated with 0.2% Triton-X-100 to disrupt the host cells and release *C. albicans* cells. Wells were scraped, the total contents of the wells were serially diluted in PBS and plated onto YPD plates for 24 h at 30°C to determine colony forming units (CFUs).

### Live-cell imaging of fungal growth

*C*. *albicans* SC5314 pre-cultured in VSM with or without lactic acid at pH 4.5 or pH 6 was washed with PBS before being resuspended in RPMI 1640 (supplemented with glutamine; Gibco) medium and 1 × 10^4^ yeast cells/well were seeded in flat-bottom 96-well plates. For oxidative stress, 1 mM H_2_O_2_ (or H_2_0 as control) was added to the culture medium. Plates were incubated in an Incucyte S×5live-cell analysis system (Sartorius) in an incubator at 37°C and 5% CO_2_. Phase contrast images at 20 × magnification were acquired at an interval of 30 min. Following imaging, hyphal growth was analysed using the NeuroTrack analysis software V. (Sartorius) as previously established [42].

### Adhesion and invasion assay

For adhesion, confluent VECs in 24-well flat-bottom plates were infected with 500 µL of 2 × 10^5^ *C. albicans* cells/mL (MOI 1) in RPMI for 1 h at 37°C and 5% CO_2_. Following one washing step in PBS, samples were fixed with 4% Histofix for 15 min at 37°C. Fungal cells were stained with calcofluor white (CFW; 10 µg/mL in 0.1 M Tris-HCl, pH 9.5; Sigma-Aldrich) and incubated at RT in the dark. After 20 min, cells were washed three times with dH_2_0 and images were taken using a Zeiss Cell Discoverer 7 microscope at 20× magnification. The number of adherent fungal cells is expressed as percentage of adhered cells of inoculum [43].

For invasion, confluent VECs in 24-well plates were infected with 500 µL of 2 × 10^5^ *C. albicans* cells/mL (MOI 1) in RPMI for 3 h at 37°C and 5% CO_2_. After washing once with PBS, samples were fixed with 4% Histofix for 15 min at 37°C, and extracellular *C. albicans* cells were stained with Concanavalin A (50 µg/mL; Alexa Fluor 647; Invitrogen) for 30 min at RT in the dark. Next, VECs were washed with PBS and permeabilized using 0.5% Triton X-100 for 5 min in the dark. Internalized hyphae were stained with CFW for 20 min in the dark, the samples were washed three times with dH_2_0 and images were taken with a Zeiss Cell Discoverer 7 microscope at 20× magnification. Results are reported as the percentage of invasive *albicans* hyphae. At least 100 hyphae per condition were counted in three independent experiments.

### Ethics statement

Inclusion of healthy volunteers was approved by both the local institutional review board in Nijmegen (CMO region Arnhem-Nijmegen, The Netherlands, no. 22992010/104) and the institutional ethics committee of Jena University Hospital (Permission no. 2207–01/08). Inclusion of volunteers and experiments were conducted following the principles expressed in the Declaration of Helsinki.

### Isolation and stimulation of peripheral blood mononuclear cells

Peripheral blood mononuclear cells (PBMCs) and Percoll monocytes were isolated from buffy coats from healthy donors after written informed consent (Sanquin Blood Bank, Nijmegen, the Netherlands or Transfusion Medicine University Hospital Jena). Due to the anonymity of donor information, we were unable to obtain specific demographic information and history of VVC from the healthy volunteer participants.

PBMCs were isolated as previously described [44]. Briefly, blood was diluted 1:1 in PBS and separated by Ficoll-Paque density gradient centrifugation (Ficoll-Paque, GE Health care). After removal of the PBMC fraction, the cells were washed twice with PBS and resuspended in RPMI+ (supplemented with 50 μg/mL gentamicin, 2 mM L-glutamine, and 1 mM pyruvate) culture medium at a final concentration of 5 × 10^6^ cells/mL. For PBMC stimulation experiments, 100 μL of 5 × 10^6^ cells/mL were seeded in a round-bottom 96-well plate and incubated with 50 μL of stimulus (RPMI+ only as a negative control, 1 × 10^6^/mL of live or thimerosal-killed *C. albicans* yeast grown in VSM with or without lactic acid) and 50 μL of medium for 24 h or 7 days [supplemented with 10% human pooled serum (HPS)]. The plates were spun down at 1400 rpm for 8 min and the collected supernatants were stored at −20°C until further analysis.

### Isolation and stimulation of polymorphonuclear cells

Polymorphonuclear cells (PMNs) were isolated from fresh EDTA blood from healthy donors after written informed consent. After the density gradient centrifugation step as described above, hypotonic lysis buffer (155 mM NH_4_Cl and 10 mM KHCO_3_) was added twice for 15 min on ice to the erythrocyte/granulocyte fraction, with a washing step in between using cold PBS, to lyse the erythrocytes. The remaining granulocytes were washed once in cold PBS, counted, and resuspended in RPMI+ at a concentration of 5 × 10^6^ PMNs/mL. For PMN stimulation experiments, 100 μL of 5 × 10^6^ PMNs/mL were seeded in a round-bottom 96-well plate and incubated with 50 μL of RPMI (only as a negative control) or 1 × 10^6^/mL of live or thimerosal-killed *C. albicans* yeast grown in VSM with or without lactic acid, and brought to a final volume of 200 μL with RPMI+ for 4 h at 37°C and 5% CO_2_. Following centrifugation at 1400 rpm for 8 min, supernatants were stored at −20°C until further analysis.

### Reactive oxygen species production assay

Reactive Oxygen Species (ROS) production was measured by oxidation of luminol (5-amino-2,3-dihydro-1,4-phtalazinedione). In brief, 100 μL of 5 × 10^6^ PMNs/mL were seeded in white flat-bottom 96-well plates and stimulated with 100 μL of either RPMI as negative control or 1 × 10^7^/mL live *C. albicans* cells (MOI 2) grown in VSM with or without lactic acid, both pre-opsonized with HPS for 30 min at 37°C and 5% CO_2_. Finally, 200 μL of luminol (1 mM in DMSO; Sigma-Aldrich) in Hank’s Balanced Salt Solution (HBSS; Gibco) and 0.5% Bovine Serum Albumin (BSA) were added to each well. All the conditions were performed in technical triplicates. Luminescence was measured every 2.23 min for 1 h at 37°C in a BioTek Synergy HTreader (Winooski, VT, USA). Data representing the relative luminescence units per second (RLU/sec) within the area under the curve (AUC) during the measuring time were plotted and analysed by using GraphPad Prism v 8.0.2.

### Monocyte isolation and in vitro induction of trained immunity

Monocytes were isolated using a hyperosmotic density gradient separation using Percoll, as previously described [45]. In brief, 150–200 × 10^6^ PBMCs were layered on top of a hyperosmotic solution [48.5% Percoll (Sigma-Aldrich), 41.5% sterile dH_2_O, and 0.16 M filter-sterilized NaCl]. After centrifugation and harvesting the interphase layer, cells were washed with cold PBS and resuspended in RPMI+ to a final concentration of 1 × 10^6^ monocytes/mL. *In vitro* induction of trained immunity was performed as previously described [46]. Briefly, 1 × 10^5^ monocytes/well were seeded for 1 h at 37°C and 5% CO_2_ in flat-bottom 96-well plates. Non-adherent cells were removed by a PBS washing step and subsequently the plates/adherent cells were incubated for 24 h with 200 μL of RPMI+ (negative control), 1 × 10^6^/mL HK *C. albicans* (grown in YPD, positive control), or 1 × 10^6^/mL thimerosal-killed *C. albicans* yeast grown in VSM with or without lactic acid, supplemented with 10% HPS. After 24 h cells were washed once with 200 μL warm PBS and incubated for 5 days in culture medium with 10% HPS and medium was changed once. After 6 days, trained cells were challenged with 200 μL RPMI (negative control), *Escherichia coli* lipopolysaccharide (LPS; 10 ng/mL), Pam3Cys (P3C; 10 μg/mL), peptidoglycan (PGN; 10 μg/mL), or 1 × 10^7^/mL HK *E. coli* (*E. coli*; ATCC 35,218) for 24 h. On day 7, plates were centrifuged at 1400 rpm for 8 min and the collected supernatants were stored at −20°C until further analysis.

### Human macrophage differentiation and infection

After PBMCs isolation, CD14-positive monocytes were selected by magnetic automated cell sorting (autoMACs) (MiltenyiBiotec). To differentiate monocytes into human MDMs (hMDMs), 1.7 × 10^7^ cells were seeded into 175 cm^2^ cell culture flasks in RPMI+ (Thermo Fisher Scientific) containing 10% heat-inactivated FBS (RPMI+FBS; Bio&SELL) and 50 ng/mL recombinant human M-CSF (ImmunoTools) and incubated for 7 days at 37 °C and 5% CO_2_. Adherent hMDMs were detached with 50 mm EDTA in PBS, and 200 μL of 2 × 10^5^ hMDMs/mL in RPMI+FBS were seeded in 96-well plates and incubated overnight before stimulation experiments were performed in serum-free RPMI medium.

### Inflammasome activation

To assess inflammasome activation, 200 μL of 2 × 10^5^ hMDMs/mL were seeded in flat-bottom 96-well plates. After 24 h, hMDMs were primed with LPS (50 ng/mL) for 2 h and subsequently stimulated with RPMI (negative control), 4 × 10^4^/well of live *C. albicans* cells (MOI 1) grown in VSM with and without lactic acid, or Nigericin (1 μM, positive control; Sigma-Aldrich) for 5 h at 37°C, 5% CO_2_. After incubation, cells were centrifuged at 250 g for 10 min and the collected supernatants were stored at −20°C until Interleukin (IL)-1β concentrations were determined by ELISA.

### Time-lapse imaging to quantify hMDM cell death

Macrophages death was assessed as previously described [47]. Briefly, 300 μL of 2 × 10^5^ hMDMs/mL were seeded in μ-Slide 8-Well (IBIDI) and incubated overnight at 37°C and 5% CO_2_. Macrophages were infected with 150 μL of 4 × 10^5^ live *C. albicans* cells/mL (MOI 1), previously grown in VSM with and without lactic acid, using RPMI containing SYTOX Green (Invitrogen) to stain non-viable macrophages. Cell death was quantified by time-lapse imaging using a Zeiss Cell Observer microscope with an integrated AxioCam 506 controlled using Zeiss Zen software. Four independent fields/well were imaged in 15 min intervals at 10× magnification for a maximal period of 24 h using the bright field channel and Alexa 488 filters. The green channel images were processed using FIJI software [40]. After conversion to binary images, SYTOX-positive cells were enumerated using the Particle Analyzer tool and macro batch analysis. The average total number of macrophages per field of view was determined by lysing macrophages using 0.1% Triton-X100 in PBS and counting SYTOX-positive macrophages.

### *C. albicans* fitc-labelling and phagocytosis capacity of PMNs

1× 10^8^/mL thimerosal-killed VSM-grown *C. albicans* cells were incubated with 0,001 mg/mL fluorescein isothiocyanate (FITC) while shaking at 4°C in the dark. After 30 min, fungal cells were centrifuged, washed three times in cold PBS, and adjusted to a final concentration of 2× 10^8^/mL using an Improved Neubauer counting chamber. 100 μL of 2 × 10^6^ PMNs/mL were seeded in flat-bottom 96-well plates and stimulated with opsonized thimerosal-killed FITC-labelled *C. albicans* (MOI 2) for 2 h. Phagocytosis of fungal cells by neutrophils was analysed by flow cytometry (Cytoflex, Beckman Coulter) and results are expressed as the percentage of internalized cells based on the mean fluorescence intensity (FI).

### Killing of fungal cells by PMNs and hMDMs

A sample of 100 μL of 1 × 10^6^ PMNs/mL or 200 μL of 2 × 10^5^ hMDMs/mL in RPMI was seeded into flat bottom 96-well plates and stimulated with live *C. albicans* grown in VSM with and without lactic acid (MOI 1), respectively, for 3 and 5 h at 37°C, 5% CO_2_. Following incubation, cells were lysed with dH_2_0, supernatants were serially diluted in PBS, and plated on YPD plates to determine *C. albicans* survival as CFU.

### Cytokine measurements

Cytokine release was quantified in frozen supernatants from VECs, PBMCs, hMDMs, and PMNs stimulation experiments using commercial enzyme-linked immunosorbent assay (ELISA) kits for Tumor Necrosis Factor (TNF), Interleukin (IL)-6, IL-1β, IL-1Ra, IL-8, IL-10, IL-17, IL-22, Monocyte Chemoattractant Protein (MCP)-1, and Interferon (IFN)-γ (Human Duoset ELISA, Bio-Techne, R&D systems, MN, USA) following the manufacturer’s instructions.

### Electron microscopy

*C*. *albicans* cells grown in VSM containing or lacking lactic acid were rapidly passed through a polycarbonate filter under mild vacuum, immediately transferred into 3 mm aluminium planchettes (0.1 mm depth), and then fixed by high pressure freezing (HPM Live µ, CryoCapCell, Le Kremlin-Bicêtre, France). Samples were freeze-substituted similar to that described by [48]. In brief, samples were transferred under liquid nitrogen into cryovials containing 1% osmium tetroxide and 0.5% glutaraldehyde in acetone and then placed in an aluminium block on a rotary shaker and covered with dry ice. After 3 h, the dry ice was removed, and the samples incubated for another hour on the shaker. Samples were subsequently washed 3 times for 5 min at RT in Epon. Ultrathin sections (60 nm) were collected on pioloform-coated 100 mesh copper EM grids, contrasted in lead citrate, and imaged with a JEOL 1400 JEM transmission electron microscope using a digital camera (ES1000W, Gatan, Ametek, Abingdon, UK). Regions of interest, containing orthogonal sections of the cell wall with clear demarcation of the underlaying plasma membrane, were sampled at random and imaged at a nominal magnification of 1000 × . Approximately 30 cells were imaged per condition. For each cell, five measurements each were taken for the inner and outer cell wall using Image-J [49].

### Measurement of cell wall chitin content

*C. albicans* SC5314 yeast cells were grown in VSM with or without lactic acid and harvested after overnight growth. Following thimerosal-fixing, cells were stained for total chitin using 20 μg/mL CFW in the dark for 15 min. Stained cells were washed twice with PBS before quantification of their fluorescence by flow cytometry using a BD Fortessa flow cytometer, as described previously [31]. The plots represent three biological replicate experiments, each of which acquired 10,000 events. As a negative control, cells were treated as above, but without the addition of CFW. Median FI was determined using FlowJo V10 software (BD Life Sciences).

### Measurement of cell wall mannan and β-glucan content

An aliquot of 5 × 10^5^ yeast cells/mL from overnight cultures in VSM±LA were fixed with 4% Histofix for 15 min at 37°C. Cells were washed and resuspended in PBS. To investigate mannan exposure, fungal cells were stained with 25 µg/mL Concanavalin A-AlexaFluor647 (Invitrogen) for 30 min at 37°C. To evaluate β-glucan exposure, fungal cells were first stained with 10 µg/mL mouse-anti-β-glucan (Biosupplies, Australia) for 30 min at 37°C, washed with PBS, and then stained with 10 µg/mL of a secondary goat-anti-mouse-AlexaFluor488 (Invitrogen) for 30 min at 37°C. Stained cells were washed once with PBS before quantification of their fluorescence by flow cytometry using a BD FACSVerse Cell Analzyer flow cytometer. As a negative control, cells were treated as above, but without the addition of any staining agent. Median FI was determined using FlowJo V10 software (BD Life Sciences).

### Fluorescence microscopy

A sample of 2 μL of CFW stained cells were pipetted on to slides with a 2% (w/v) agarose pad and a cover slide placed on top. Images were taken using DeltaVision Elite fluorescence microscope fitted with a pco.edge sCMOS camera and a Lumencor LED light source. A 100× objective was used with a numerical aperture of 1.40. Z-stacks consisting of 50 images were taken using a UV filter set. Using Image J, the Z-stacks were converted into maximum intensity projections to generate a single image.

### Statistical analysis

Graphs show the mean values of at least two biological replicates. Statistical analysis was performed using GraphPad Prism v 8.0.2. Significance was calculated using either Wilcoxon matched-pairs signed-rank test (paired, non-parametric), two-way Analysis of Variance (ANOVA) or Student‘s t-tests and is indicated by asterisks in the figures (* = *p* < 0.05; ** = *p* < 0.01; *** = *p* < 0.001; **** = *p* < 0.0001).

## Results

### Lactic acid affects *C. albicans* virulence traits

A vaginal simulative medium (VSM) was used to mimic conditions in the human vaginal milieu *in vitro* and to systematically investigate how lactic acid (LA) affects *C. albicans* biology and interactions with the host at the vaginal epithelial barrier. Lactic acid-depleted medium (VSM-LA) was used as a control condition.

**Figure 1.**
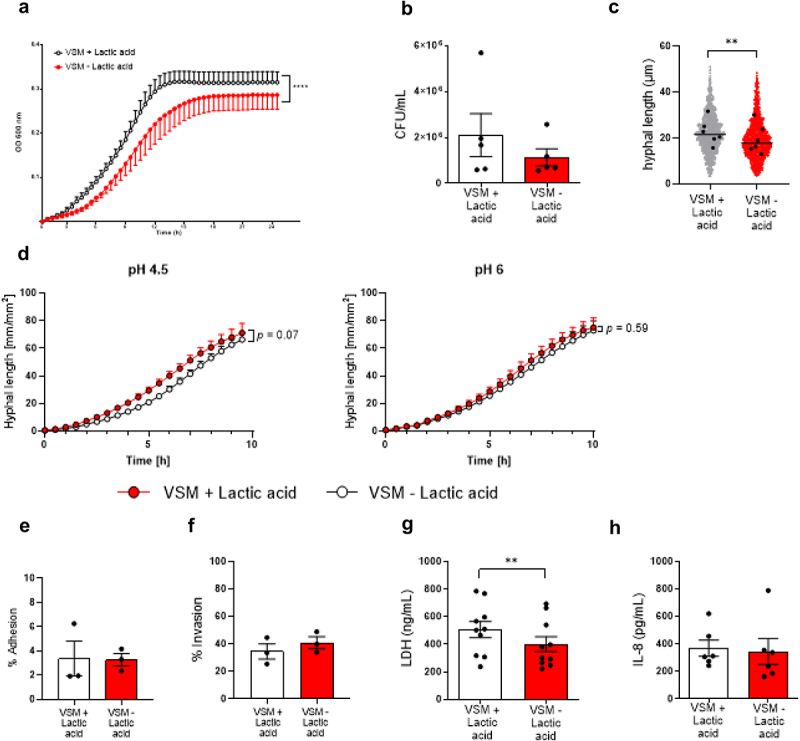
Growth in lactic acid-containing vaginal simulative medium (VSM) affects *Candida albicans* virulence traits. The virulence traits of *C. albicans* that were grown overnight in VSM with and without lactic acid, respectively, were evaluated in RPMI medium. a) Growth of *C. albicans* was measured by determining the optical density (OD) at 600 nm at 37°C for 24 h. b) *C. albicans* colony forming units (CFUs) associated with vaginal epithelial cells (VECs) after 24 h of infection at 37°C and 5% CO_2_. c) *C. albicans* hyphal length after 3 h of incubation at 37°C. Statistical analysis was performed using a two-way ANOVA. d) Growth of *C. albicans* pre-cultured in VSM at pH 4.5 and pH 6 was compared by monitoring hyphal length (mm/mm^2^) over time at 37°C and 5% CO_2_ for 10 h. Statistical analysis was performed using a two-way ANOVA with a Holm-Šídák’s multiple comparisons test. e) Adhesion of *C. albicans* cells to VECs after 1 h of infection at 37°C and 5% CO_2_ (shown as % of adherent cells compared to the initial inoculum). Data are the mean of *n* = 3, pooled from 3 independent experiments. f) Invasion of VECs by *C. albicans* after 3 h of infection at 37°C and 5% CO_2_ (shown as % of *C. albicans* hyphae that invaded VECs of total hyphae present). Data are the mean of *n* = 3, pooled from 3 independent experiments. g) Quantification of VEC damage as released lactate dehydrogenase (LDH) after 24 h of infection at 37°C and 5% CO_2_. H) IL-8 release was measured in the cell culture supernatant of *C. albicans* infected VECs after 24 h. Statistical analysis was performed using a two-tailed Wilcoxon matched pairs signed-rank test. Results are shown as the mean ± SEM of at least 2 independent experiments. Statistical significance is shown as ** = *p* < 0.01 and **** = *p* < 0.0001.

The presence of lactic acid in VSM (VSM+LA) was associated with increased *C. albicans* growth (*p* < 0.0001; Figure 1a, which correlated with a trend for higher numbers of *C. albicans* colony forming units (CFUs) on vaginal epithelial cells (VECs) after infection (Figure 1b). Hyphae of *C. albicans* grown in VSM+LA were longer in comparison to fungal cells grown in VSM-LA (*p* = 0.0313; Figure 1c). Since lactate can be used as a carbon source by *C. albicans*, we also tested the effect of VSM with and without lactic acid at pH 6 where the balance is shifted towards a greater lactate:lactic acid ratio. Live-cell imaging revealed that both at pH 6 the presence of lactic acid was not associated with increased *C. albicans* hyphal growth, whereas a trend was observed at pH 4.5 (Figure 1d). *C. albicans* cells grown in VSM at pH 4.5 showed a similar ability to adhere to and invade VECs (Figure 1e,f). In line with the production of longer hyphae, the ability of *C. albicans* to cause epithelial cell damage when grown in VSM+LA was increased in comparison to that caused by fungal cells grown in VSM-LA (*p*  = 0.0039; Figure 1g). Nevertheless, release of the crucial neutrophil chemoattractant IL-8 by VECs infected with *C. albicans* grown under the two conditions was comparable (Figure 1h).

### Lactic acid affects *C. albicans* cell wall architecture

The subtle changes in *C. albicans* pathogenicity traits hint towards altered fungal biology.

**Figure 2.**
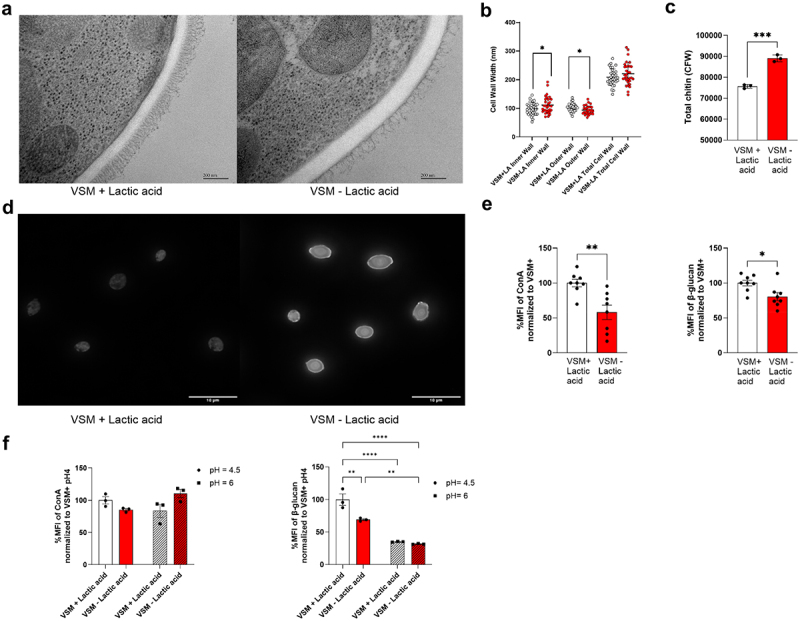
Lactic acid affects *Candida* cell architecture.

The *C. albicans* cell wall is a highly dynamic structure that responds to the environment [32]. Therefore, we investigated whether lactic acid in VSM affected *C. albicans* cell wall architecture. Using transmission electron microscopy (TEM), the width of the inner and outer individual layers of the cell wall was measured and using flow cytometry the levels of mannan and β-glucan exposure were quantified. Evaluation of TEM images showed that growth in VSM+LA altered *C. albicans* cell wall structure (Figure 2a). Specifically, the mannan fibrils in the outer layer were denser and more organized compared to the cell walls of *C. albicans* cells grown in VSM-LA (Figure 2a). The presence of lactic acid in VSM led to a thinner inner layer (*p* = 0.0478; Figure 2b) and thicker outer cell wall compared to the control cells (*p* = 0.0126; Figure 2b). In line with these observations, fungal cells grown in VSM+LA displayed a decrease in the total chitin content, as shown both by flow cytometry (*p* = 0.0002; Figure 2c) and fluorescence microscopy data (Figure 2d). In a separate set of experiments, we observed that both mannan and β-glucan exposure were increased when fungal cells were grown in VSM+LA (mannan: *p* = 0.0029; β-glucan: *p* = 0.0141; Figure 2e). Consistent with previous findings [50], *C. albicans* grown in VSM+LA showed greater β-glucan exposure at pH 4.5 compared to at pH 6. Yet, only at pH 4.5 the presence of lactic acid further enhanced β-glucan exposure (Figure 2f). These changes in the *C. albicans* cell wall, as well as the pathogen-associated molecular patterns (PAMPs) it harbours, could result in changes in immune recognition. Therefore, we followed up on this observation by systematically assessing interactions with different immune cell types.

a) Transmission electron micrographs (TEM) showing the cell walls of wild type *Candida albicans* SC5314 cells grown in vaginal simulative medium (VSM) with and without lactic acid (LA). b) Quantification of the thickness of the inner and outer layers of the *C.* *albicans* SC5314 cell wall using Image-J from TEM images such as those shown in panel A. Means and standard deviations from images of cells (*n* = ~30 cells; 5 independent inner and outer cell wall measurements are taken from each cell) from three independent replicate experiments are shown. c) Analysis of chitin levels in *C.* *albicans* SC5314 cells by flow cytometry of calcofluor white (CFW)-stained cells grown in VSM+LA (white) or VSM-LA (red). Median fluorescence intensities (MFIs) for both cell populations are shown.

d) Three-dimensional fluorescence images acquired using Delta Vision microscopy (100× objective). Cells were grown in VSM in the presence (left panel) or absence of lactic acid (right panel), cell wall chitin stained with 20 µg/ml CFW and then subjected to fluorescence imaging: scale bars, 10 μm. e) Analysis of mannan (ConA) and β-glucan (anti-β-glucan) exposure of VSM±LA at pH 4.5 grown *C.* *albicans* SC5314 yeast cells measured by flow cytometry. f) Analysis of mannan (ConA) and β-glucan (anti-β-glucan) exposure of *C.* *albicans* SC5314 yeast cells grown in VSM±LA at pH 4.5 compared to at pH 6 as measured by flow cytometry. MFIs were normalized to the corresponding VSM+LA control and are displayed as mean ± SEM with dots representing single cultures. Data presented in panel b, c, and e were analysed using Student‘s t-tests to assess significant differences. Data presented in panel f were analysed using two-way ANOVA with Holm-Šídák’s multiple comparisons test. Statistical significance is shown as * = p < 0.05, ** = p < 0.01, and *** = p < 0.001.

### Growth in the presence of lactic acid increases *C. albicans* immunogenicity

**Figure 3.**
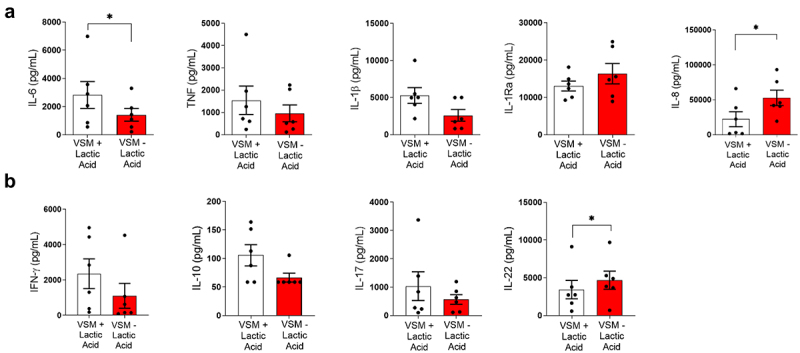
Innate and adaptive cytokine profile in human peripheral blood mononuclear cells (PBMCs) upon stimulation with *Candida albicans* cells grown in lactic acid-depleted vaginal simulative medium (VSM). a) PBMCs from healthy volunteers were stimulated with live *C*. *albicans* grown either in VSM with or without lactic acid for 24 h at 37°C and 5% CO_2_. Levels of IL-6, TNF, IL-1β, IL-1Ra, and IL-8 in cell culture supernatants were measure by ELISA. b) IFN-γ, IL-10, IL-17, and IL-22 levels were measured in supernatant of PBMCs stimulated for 7 days with thimerosal-killed *C. albicans* cells grown in VSM with or without lactic acid. Statistical analysis was performed using a two-tailed Wilcoxon matched pairs signed-rank test. Results are shown as the mean ± SEM pooled from at least two independent experiments. Statistical significance is shown as * = *p* < 0.05.

To evaluate how the altered cell wall by growth in a lactic acid rich vaginal simulative environment impacts overall immunogenicity, cytokine release by peripheral blood mononuclear cells (PBMCs) was assessed. PBMCs released higher amounts of IL-6 in response to live *C. albicans* grown in VSM+LA (*p* = 0.0312; Figure 3a). Overall, a trend towards higher induction of TNF and IL-1β was observed (Figure 3a). Interestingly, we observed lower concentrations of IL-8 in response to *C. albicans* grown in VSM+LA medium (*p* = 0.0312; Figure 3a). T-cell cytokine responses quantified after 7-day stimulation of PBMCs with thimerosal-killed *C. albicans* cells grown in VSM+LA revealed an overall trend towards higher levels of IFN-γ, IL-10, and IL-17 (Figure 3b). However, a lower concentration of IL-22 was observed in response to *C. albicans* cells grown in VSM+LA (*p* = 0.0312; Figure 3b).

### Macrophages show increased cytokine responses but diminished clearance to *C. albicans* grown in the presence of lactic acid

**Figure 4.**
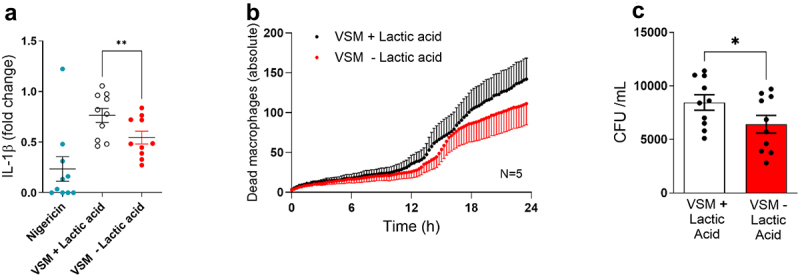
Macrophage responses upon stimulation with *Candida albicans* cells grown in lactic acid-rich vaginal simulative medium (VSM). a) Inflammasome activation was assessed by measuring IL-1β levels in the cell culture supernatant from lipopolysaccharide (LPS)-primed human monocyte-derived macrophages (hMDMs) that were stimulated for 5 h at 37°C and 5% CO_2_with either Nigericin (positive control) or *C. albicans* cells grown in VSM with or without lactic acid (LA). Results are presented as fold change of *C. albicans* grown in yeast extract-peptone-dextrose (YPD) medium. b) Macrophage death was assessed at 37°C and 5% CO_2_by quantifying SYTOX green release by hMDMs stimulated with *C. albicans* cells grown overnight in VSM±LA. Data are shown as the mean of 5 different donors. Statistical analysis was performed using a two-way ANOVA with Tukey’s multiple comparisons test. c) hMDMs killing capacity is shown as *C. albicans* colony forming units (CFUs) after 5 h of stimulation at 37°C and 5% CO_2_. Statistical analysis was performed using a test two-tailed Wilcoxon matched pairs signed-rank test. Results are shown as the mean ± SEM of at least 2 independent experiments.

Given the crucial role of NLRP3 inflammasome-mediated IL-1β responses during vulvovaginal candidiasis (VVC) [10,12], we evaluated macrophage responses to *C. albicans* cells grown in VSM with or without lactic acid. Because inflammasome activation in macrophages is a two-step process, human monocyte-derived macrophages (hMDMs) were first primed with lipopolysaccharide (LPS) and then challenged with live *C. albicans* cells grown in VSM±LA. Macrophages released more IL-1β in response to *C. albicans* cells grown in VSM+LA (*p* = 0.0073; Figure 4a). In addition, we monitored macrophage death in the presence of live *C. albicans* cells. An overall tendency was observed for reduced macrophage survival when interacting with VSM+LA grown *C. albicans* in comparison to *C. albicans* grown in VSM-LA (Figure 4b). However, despite showing an increase in 4 out of 5 donors, the overall effect was not statistically significant due to inter-individual variation (Supplementary Figure S1). Consistent with reduced macrophage viability, macrophages were less efficient at killing *C. albicans* grown in VSM+LA, as reflected by higher recovered CFU numbers (*p* = 0.0273; Figure 4c).

### Induction of trained immunity by *C. albicans* grown in the presence of lactic acid

*C*. *albicans* has been shown to confer long-term increased responsiveness of innate immune cells (a *de-facto* innate immune memory termed trained immunity) [17]. A dysregulated induction of the trained immunity program could explain the hyperresponsiveness of recurrent VVC (RVVC) patients to *C. albicans*. Therefore, we investigated whether *C. albicans* grown in VSM including lactic acid altered its ability to induce trained immunity.

**Figure 5.**
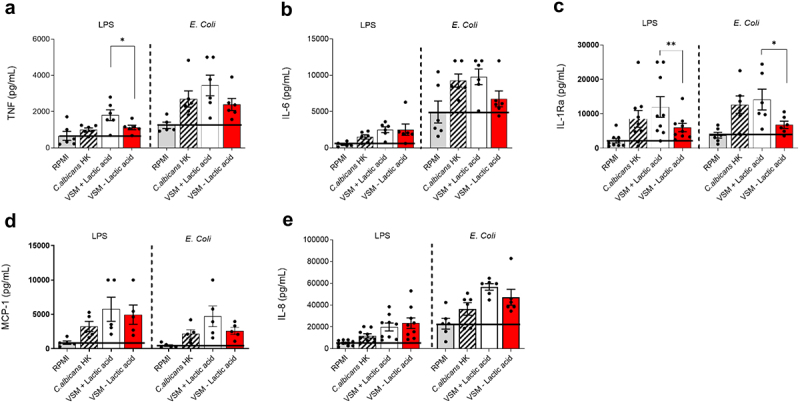
Induction of trained immunity by *Candida albicans* grown in the presence of lactic acid. a-e) Following the *in vitro* trained immunity protocol, monocytes isolated from healthy buffy coats were stimulated with either heat-killed (HK) *C. albicans* (positive control, grown in YPD) or *C. albicans* cells grown overnight in vaginal simulative medium (VSM) in the presence or absence of lactic acid. After 24 h, the training stimulus was washed, and cell were let to rest for 5 days. On day 6, cells were restimulated for 24 h with either lipopolysaccharide (LPS) or HK *Escherichia coli*. Levels of TNF (A), IL-6 (B), IL-1Ra (c), MCP-1 (d), and IL-8 (e) were measured in cell culture supernatants by ELISA. Statistical analysis was performed using a two-tailed Wilcoxon matched pairs signed-rank test. Results are shown as the mean ± SEM pooled from at least 3 independent experiments. Statistical significance is shown as * = *p* < 0.05 and ** = *p* < 0.01.

Monocytes were trained with heat-killed (HK) *C. albicans* grown in yeast extract-peptone-dextrose (YPD; positive control) or thimerosal-killed *C. albicans* grown in VSM±LA. Induction of trained immunity was assessed after 6 days by quantifying cytokine response to re-stimulation with HK *Escherichia coli* and different synthetic PRR ligands: Lipopolysaccharide (LPS), Pam3Cys (P3C), and Peptidoglycan (PGN). Culturing *C. albicans* in VSM in the presence or absence of lactic acid induced trained immunity differentially. Specifically, monocytes exposed to lactic acid-rich grown *C. albicans* showed an overall increased training ability, reflected in significantly higher TNF responses towards LPS restimulation (*p* = 0.0312; Figure 5a) and increased TNF production after *E. coli*, P3C, and PGN restimulation (Figure 5a and Supplementary Figure S2) as compared to monocytes trained with *C. albicans* cells grown in VSM without lactic acid. Likewise, monocytes trained with *C. albicans* cells grown in VSM+LA produced higher amounts of IL-6, particularly upon P3C re-stimulation (*p* = 0.0312; Figure 5b and Supplementary Figure S2). In line with the increased pro-inflammatory response of monocytes trained with *C. albicans* cells grown in VSM containing lactic acid, we observed higher amounts of anti-inflammatory IL-1Ra in response to all of the secondary stimuli tested (LPS: *p* = 0.0078; *E. coli*: *p* = 0.0312; PNG: *p* = 0.0478; P3C: *p* = 0.0312; Figure 5c and Supplementary Figure S2). Lastly, release of the chemoattractant MCP-1 and IL-8 was not altered when monocytes were trained with *C. albicans* cells grown in VSM±LA and restimulated with various PRR ligands (Figure 5d,e and Supplementary Figure S2).

### Neutrophils show dysfunctional phenotype upon interacting with *C. albicans* grown in lactic acid-rich medium

**Figure 6.**
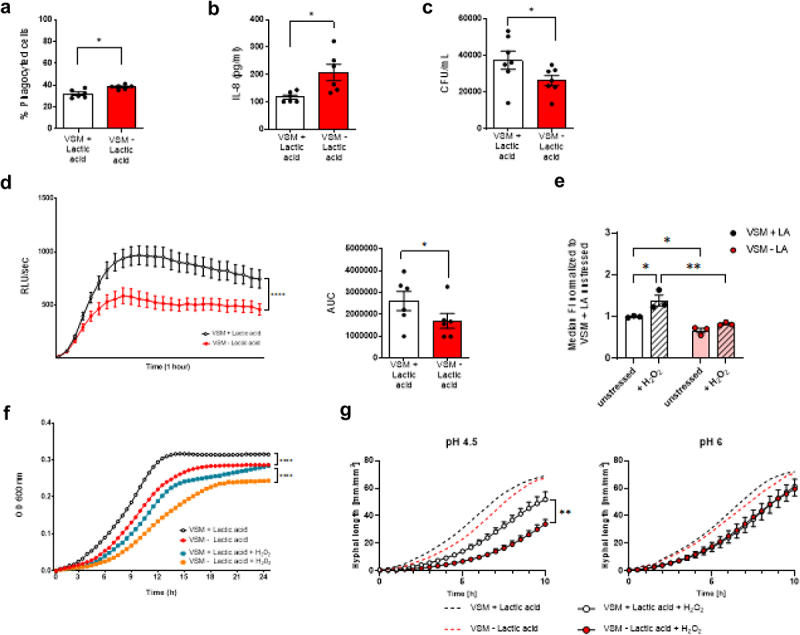
Neutrophils show dysfunctional phenotype upon interaction with *Candida albicans* cells grown in lactic acid-rich vaginal simulative medium (VSM). Neutrophils isolated from healthy volunteers were stimulated with live and thimerosal-killed *C.albicans* cells grown in VSM either with or without lactic acid (LA) to assess their cellular response. a) Neutrophil phagocytosis capacity is expressed as % of FITC-positive cells after 2 h stimulation with thimerosal-killed FITC-labelled *C. albicans*. b) Neutrophils were stimulated with live *C. albicans* grown either in VSM±LA for 4 h at 37°C. Levels of IL-8 in cell culture supernatants were measured by ELISA. c) Killing capacity is shown as colony forming units (CFUs) after 3 h of stimulation at 37°C CO_2_ with live *C. albicans* cells. d) Reactive oxygen species (ROS) production in response to live *C*. *albicans* during a 1 h timeframe at 37°C is reported both as time course [relative luminescence units (RLU)/sec, left panel] and area under curve (AUC, right panel). Time course was assessed for statistical differences between the two tested conditions by a two-way ANOVA and AUC means (right panel) were compared using the two-sided Wilcoxon signed rank test. e) The *C. albicans* CTA1p-GFP reporter strain was grown overnight in VSM with or without lactic acid. After washing, cells were exposed either to H_2_0 or 2 mM H_2_0_2_ for 2 h at 37°C and fluorescence was quantified by flow cytometry. The mean fluorescence intensities (MFIs) were normalized to the corresponding VSM with lactic acid control and results are displayed as mean ± SEM with dots representing single cultures. Statistical analysis was performed using a two-way ANOVA with Holm-Šídák’s multiple comparisons test. f) Growth of *C. albicans* cells grown overnight in the tested VSM conditions were monitored in RPMI by determining the optical density (OD) at 600 nm at 37°C for 24 h in the presence or absence of 10 mm H_2_0_2_. Statistical analysis was performed using a two-way ANOVA. Statistical analysis was performed using a test two-tailed Wilcoxon matched pairs signed-rank test. g) Growth of *C. albicans* pre-cultured in VSM at pH 4.5 and pH 6 were monitored in RPMI by determining the optical density (OD) at 600 nm at 37°C for 24 h in the presence or absence of 1 mM H_2_0_2_. Statistical analysis was performed using a two-way ANOVA with a Holm-Šídák’s multiple comparisons test.Results are shown as the mean ± SEM of at least 2 independent experiments. Statistical significance is shown as * = *p* < 0.05, ** = *p* < 0.01, and **** = *p* < 0.0001.

Given the central role of neutrophils in VVC pathogenesis [51], we investigated their function in response to *C. albicans* cells grown either in lactic acid-rich or lactic acid-depleted VSM. Neutrophils phagocytosed thimerosal-killed *C. albicans* cells grown in lactic acid-rich VSM with lower efficiency (*p*  = 0.0312; Figure 6a). In line with this, neutrophils released lower IL-8 amounts upon stimulation with *C. albicans* grown in VSM+LA compared to the VSM-LA condition, suggesting compromised activation (*p* = 0.0312; Figure 6b). In addition, *C. albicans* grown in lactic acid-rich conditions were less efficiently killed by PMNs after 3 h (*p*  = 0.0469; Figure 6c). Reactive oxygen species (ROS) are one of the main antimicrobial effector mechanisms of neutrophils, yet also a major driver of immunopathology by causing damage to host tissues [52]. Thus, we assessed ROS release upon infection with serum-opsonized live *C. albicans* cells grown either in VSM containing or lacking lactic acid. Interestingly, when exposed to *C. albicans* cells grown under lactic acid rich conditions neutrophils released more ROS (*p* = 0.0312; Figure 6d). Given that the oxidative burst induced by phagocytic cells is a major mechanism for *C. albicans* killing [53], we investigated how *C. albicans* growth in VSM containing or lacking lactic acid impacts oxidative stress resistance using a reporter strain where green fluorescent protein (GFP) expression is under the control of the promotor of the catalase (CTA1) gene, under control of the actin (ACT1) promotor or GFP lacking a promotor sequence (promotor-less control) (Supplementary Figure S3) [41]. When *C. albicans* cells were grown in VSM+LA, an increased activity of the catalase promotor was observed (*p* = 0.0321; Figure 6e, white empty bar) compared to cells grown in VSM-LA (Figure 6e, red empty bar). Following exposure to hydrogen peroxide (H_2_O_2_; Figure 6e, white striped bar), *C. albicans* cells grown in VSM+LA showed even more increased catalase promotor activity (*p* = 0.0201; Figure 6e, white empty bar), whereas *C. albicans* grown in VSM-LA did not show a significant response to H_2_O_2_ (Figure 6e, red bars). Comparing VSM±LA growth conditions in the context of H_2_O_2_ oxidative stress highlights that VSM+LA grown *C. albicans* cells strongly increased catalase promoter activity compared to VSM-LA grown cells (*p* = 0.0028; Figure 6e, white and red striped bar). In line with this, exposure to 10 mm H_2_O_2_ compromised more drastically *C. albicans* growth when pre-cultured in lactic acid-depleted VSM compared to lactic acid-rich VSM (*p* < 0.0001; Figure 6f). However, when comparing VSM cultures at pH 4.5 and at pH 6, the absence of lactic acid was only associated with a poorer resistance to oxidative stress at pH 4.5 (Figure 6g, Supplementary Figure S4).

## Discussion

RVVC is a complex disease resulting from the cumulative effects of *Candida albicans* pathogenicity, the vaginal host environment, and immune responses [54]. Previous research has identified host factors like oestrogen, heparan sulphate, and human albumin as contributors to RVVC by enhancing *Candida* pathogenicity and promoting dysfunctional vaginal inflammation [55–57]. In the present study, we show that lactic acid in the vaginal niche may be another factor that diverges immune responses to *C. albicans* during RVVC. By comparing vaginal simulative medium (VSM) containing lactic acid with lactic acid-depleted VSM, we report that adaptation to lactic acid in a vaginal environment context increases fungal virulence and in parallel induces cell wall remodelling characterized by increased exposure of pathogen-associated molecular patterns (PAMPs) like mannan and β-glucan. Overall, these lactic acid-driven changes are associated with an increased capacity to stimulate cytokine responses and induce trained immunity, with the exception of a compromised neutrophil-mediated fungal clearance irrespective of enhanced oxidative responses.

Pathogenicity is an essential feature of *C. albicans* that allows it to acquire nutrients, overcome host nutritional immunity, and persist within the host [58]. We found increased vaginal epithelial cell damage when *C. albicans* SC5314 was grown in lactic acid-rich vaginal simulating conditions. Interestingly, *C. albicans* isolates from symptomatic women were found to exhibit increased pathogenicity features, compared to isolates from asymptomatic women [15,59]. However, in most women *C. albicans* does not inflict damage to host tissues. We only tested one *C. albicans* strain, the highly virulent blood isolate SC5314 [39]. In future studies, it would be important to include different *C. albicans* strains from symptomatic and asymptomatic women, particularly as strains exhibit varying tolerances to lactic acid [60]. In this context, bacteria dominating the vaginal microbiota such as lactobacilli are important competitors with *C. albicans* [26] and physiological concentrations of vaginal lactic acid, both in the D and L isomers, promote vaginal health [22,61,62], by inducing anti-inflammatory responses [63] and enhancing epithelial barrier integrity through increased expression of tight junction genes [64]. Interestingly, other organic compounds, such as acetic acid, exert stronger than lactic acid effects against *C. albicans* cells, and modulate their tolerance to antifungal agents [35,65]. Thus, the synergistic effects of the diverse metabolites present in the vaginal niche likely play a critical role in maintaining vaginal health by regulating both host tolerance and *Candida* virulence. Further research assessing the mechanisms by which vaginal metabolites inhibit growth of *Candida* species could provide valuable insights for improving the management of VVC.

We observed that *C. albicans* undergoes drastic cell wall remodelling in the presence of lactic acid. Mannans serve as an important PAMP recognized by the monocytes, but can also mask immune recognition of β-glucan in macrophages [66]. In *C. albicans* grown in lactic acid-rich VSM, exposure of mannans and the highly immunogenic cell wall polysaccharide β-glucan was increased. Similar to what we observed, acidic environments have consistently been observed to promote β-glucan exposure, leading to more potent innate immune responses [50]. We observed that at low pH value the presence of lactic acid further increases β-glucan exposure. This is in contrast to previous work showing that lactate induces immune evasion through β-1,3 glucan masking [31], underscoring that lactic acid can differentially impact cell wall architecture depending on the environmental context. For this reason, an important contributing factor to contrasting findings in cell wall remodelling may be the balance of undissociated lactic acid *versus* lactate at different pH levels. In line with higher β-glucan exposure in VSM supplemented with lactic acid at pH 4.5, β-glucan unmasking has been observed specifically in *C. albicans* isolates from symptomatic VVC patients [30]. The mannan and β-glucan exposure impacts recognition capacity by an extensive range of C-type lectin receptors including dectin-1, dectin-2, the mannose receptor (MR), DC-SIGN, and Mincle [8,67], all capable of driving potent inflammatory responses. In line with increased PAMP exposure and pathogenicity, macrophages showed higher IL-1β release in response to *C. albicans* grown in lactic acid-rich VSM. Likewise, PBMC stimulations showed an overall trend towards increased immunogenicity of *C. albicans* grown in the VSM rich in lactic acid. This subtle trend included key cytokines associated with RVVC such as IL-1β, IL-6, and IL-17 [10,13,68,69]. However, strikingly, *C. albicans* cells grown in VSM with lactic acid stimulated less IL-8 and IL-22 release by PBMCs [70]. Interestingly, an important role of IL-22 has been suggested by a recent study showing that patients with polymorphisms in the *SIGLEC15* gene associated with an increased susceptibility to RVVC produce higher IL-22 amounts [71]. On the other hand, IL-22 and its associated pathways were shown to mediate resistance to RVVC [72,73]. Overall, *C. albicans* grown in lactic acid-rich VSM led to increased responsiveness, which may lead to hyperinflammation in the context of vaginal candidiasis. This argues that lactic acid in the vaginal milieu distorts the interaction of *C. albicans* with cells of the immune system.

Neutrophils that are recruited by IL-8 play a major role in regulating the host response during *C. albicans* infection [52] both through oxidative and non-oxidative mechanisms. In addition to the reduced IL-8 release induced by *C. albicans* cells grown in the presence of lactic acid, neutrophils exhibited compromised phagocytosis and killing capacity of these cells. Interestingly, an enhanced neutrophil reactive oxygen species (ROS) release was observed. Nevertheless, *C. albicans* grown in VSM rich in lactic acid was more resistant to oxidative stress, as we observed increased levels of catalase gene expression and a consequent improved growth in the presence of H_2_O_2_. This could suggest that lactic acid induces predictive adaptation [74,75], in terms of catalase promotor activity at baseline conditions that helps *C. albicans* to cope better with future oxidative stresses. The increased ROS induction may be linked to the enhanced β-glucan exposure, which is known to drive ROS release by neutrophils [76].

Interestingly, we observed a lower IL-8 release by neutrophils in response to *C. albicans* grown in lactic acid-rich VSM, which could hint to a compromised neutrophil viability. The compromised uptake and killing of *C. albicans* may be explained by a different cell wall architecture, particularly the altered distribution of mannan fibrils in the *C. albicans* cell wall and lower chitin content. These results differ from those of Sheth and co-workers [77] who observed reduced neutrophil phagocytosis and killing activity of *C. albicans* mutant cells defective in glycosylation. However, in our study the fungal cells were grown under vaginal simulative conditions, whereas Sheth and co-workers grew *C. albicans* in Sabouraud broth. Future studies in RVVC patients are needed to evaluate the importance of our *in vitro* observation that neutrophil-mediated killing of *C. albicans* grown in lactic acid-rich VSM is compromised, since factors specific to the vaginal niche such as heparan sulphate and perinuclear anti-neutrophil cytoplasmic antibodies (pANCA) that impair neutrophil function [57,78] was not assessed in our *in vitro* model. Models using organoid [79] or organ-on-chip technology [80] in which neutrophils, vaginal epithelial cells, microbiota members, and other vaginal factors are simultaneously included should be considered in future studies to model the overall impact of lactic acid on the complex host-pathogen interplay during VVC.

*C*. *albicans*, and particularly its fungal cell wall component β-glucan, induce long-term metabolic and functional reprogramming of innate immune cells, thus conferring protection to subsequent challenge by potentiating pro-inflammatory cytokine responses [17]. When investigating the induction of trained immunity by *C. albicans* grown in a vaginal simulative environment, we observed that monocytes pre-exposed to *C. albicans* grown in VSM rich in lactic acid displayed a higher responsiveness to restimulation. This observation supports: 1) a central role for dysregulated innate immunity under vaginal simulative conditions [51], and 2) lactic acid in the vaginal environment may not only affect *C. albicans* biology, but also impact the ability of innate cells to maintain a phenotype that could mediate a pro-inflammatory state. The inappropriate induction of a trained immunity phenotype might underlie the chronic inflammatory state in RVVC patients. Corroborating this hypothesis, we have previously shown that PBMCs from RVVC patients respond with more potent responses of the innate cytokine TNF [81]. This comes in addition to *C. albicans* virulence mechanisms, which activate inflammatory responses [54]. Notably, research investigating mucosal immunity has shown that epithelial stem cells, as well as circulating immune cells, are capable of prolonged innate memory [19]. This enhanced epithelial inflammatory memory has been suggested by Cassone to play a key role in recurrent *C. albicans* infections at the vaginal mucosa [82] and future studies in the context of RVVC should expand on the induction of innate immune memory at the vaginal mucosa.

In conclusion, we demonstrate that the specific presence of lactic acid in a vaginal simulative environment modulates *C. albicans* pathogenicity and its cell wall. This leads to subsequent pro-inflammatory immune responses to *C. albicans* and dysfunctional clearance by macrophages and neutrophils. These findings strengthen the concept that specific features of the vaginal microenvironment negatively impact the finely-tuned interplay between the fungus, host, and microbiota that is dysregulated during RVVC. Our data furthers the knowledge from an immunological point of view on why immune responses in the context of RVVC diverge from their protective nature in other niches. Additional studies under vaginal simulative conditions aimed at characterizing the role of other metabolites and synergistic immune mediators involved in inflammation are needed to better understand the pathophysiology and therapeutic approaches for the treatment of VVC and RVVC.

## Supplementary Material

Supplementary Figure 3 after revision.tif

Supplementary figure S4.png

Supplementary Figure 1 after revision.tif

Supplementary Figure 2 after revision.tif

## Data Availability

The source data of this study are openly available on figshare at https://doi.org/10.6084/m9.figshare.26779465
