# Supplementary figures and images for: Lactic acid in the vaginal milieu modulates the Candida-host interaction
Source: Virulence. 2025 Jan 22;16(1):2451165. doi: 10.1080/21505594.2025.2451165 (PMC11760238; doi:10.1080/21505594.2025.2451165)

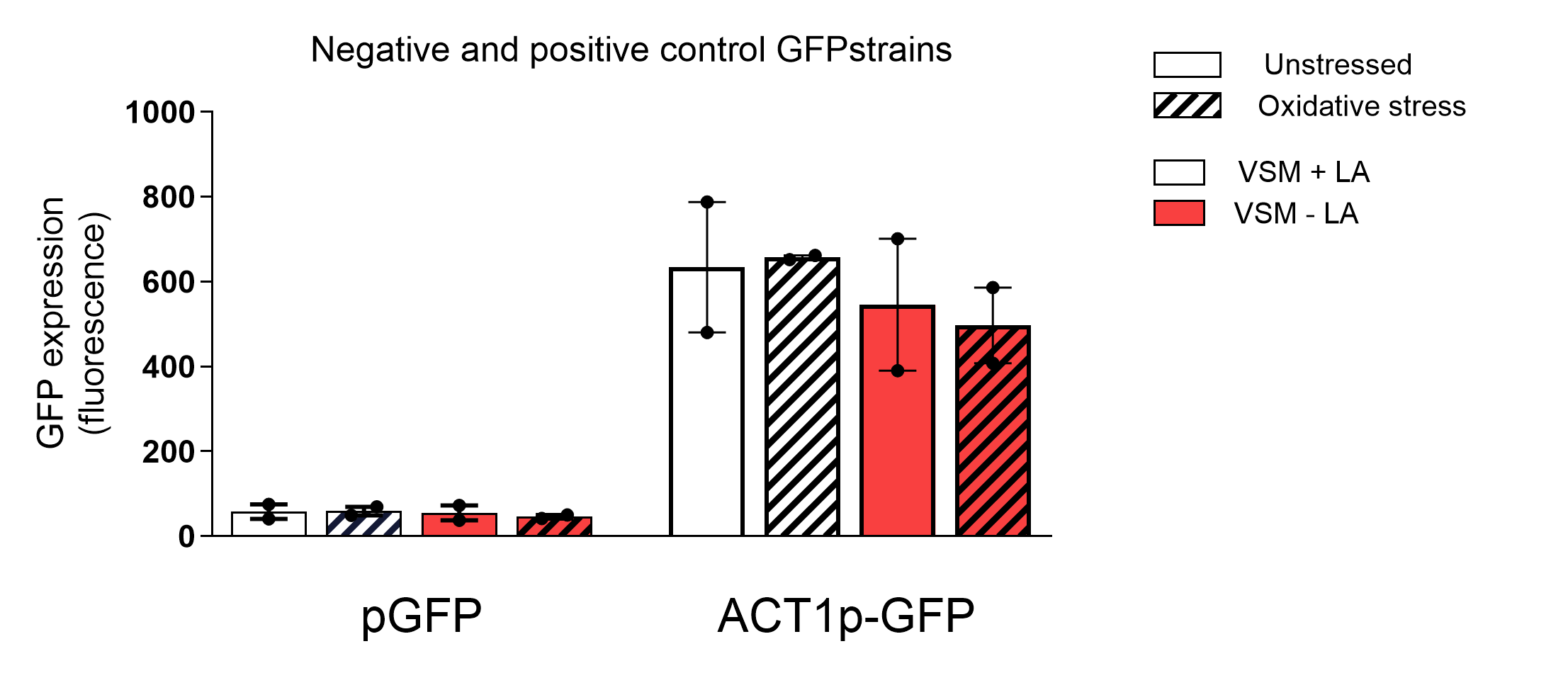

Supplement: Supplementary Figure 3 after revision.tif [file KVIR_A_2451165_SM9633.tif]

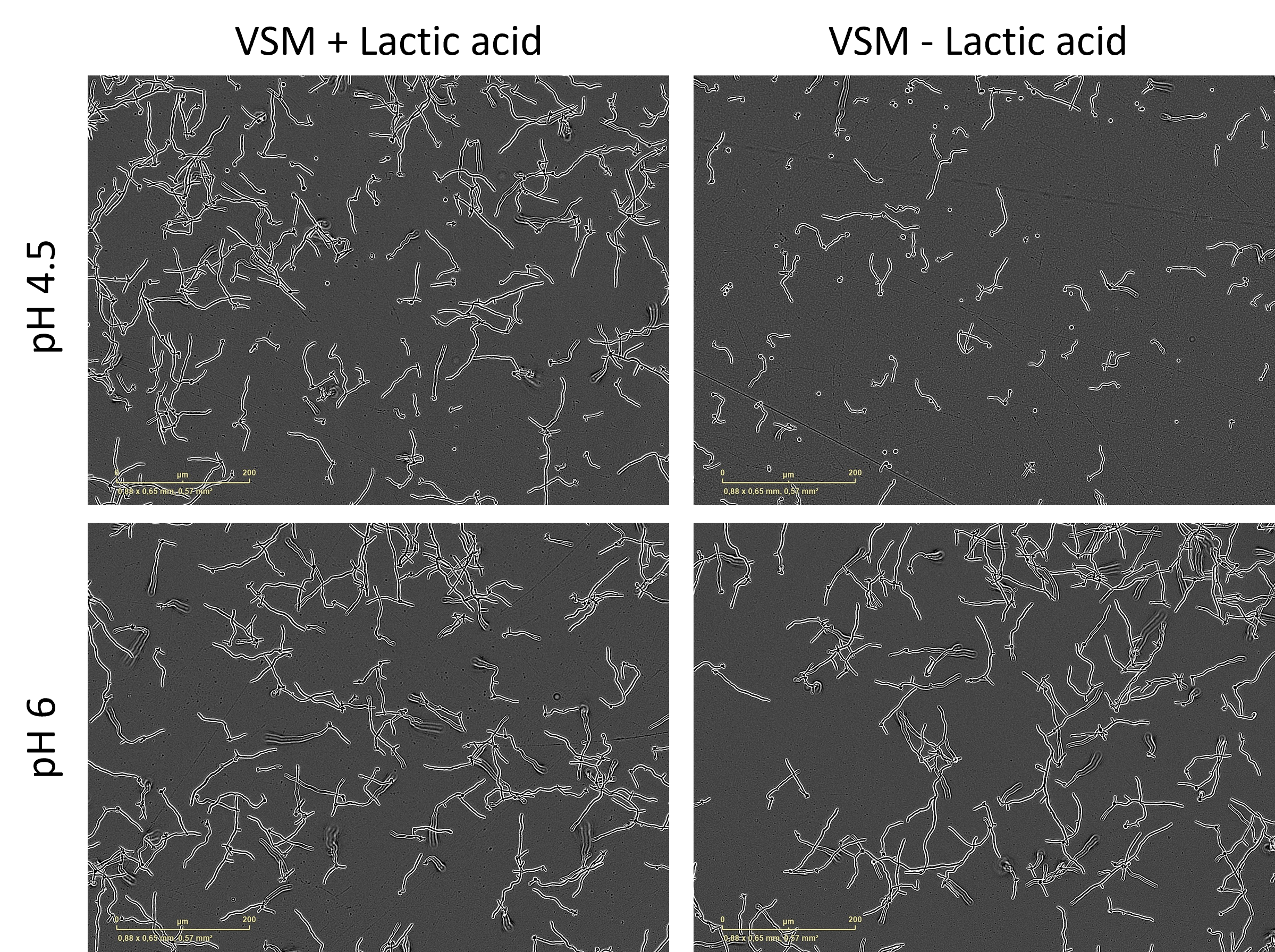

Supplement: Supplementary figure S4.png [file KVIR_A_2451165_SM9632.png]

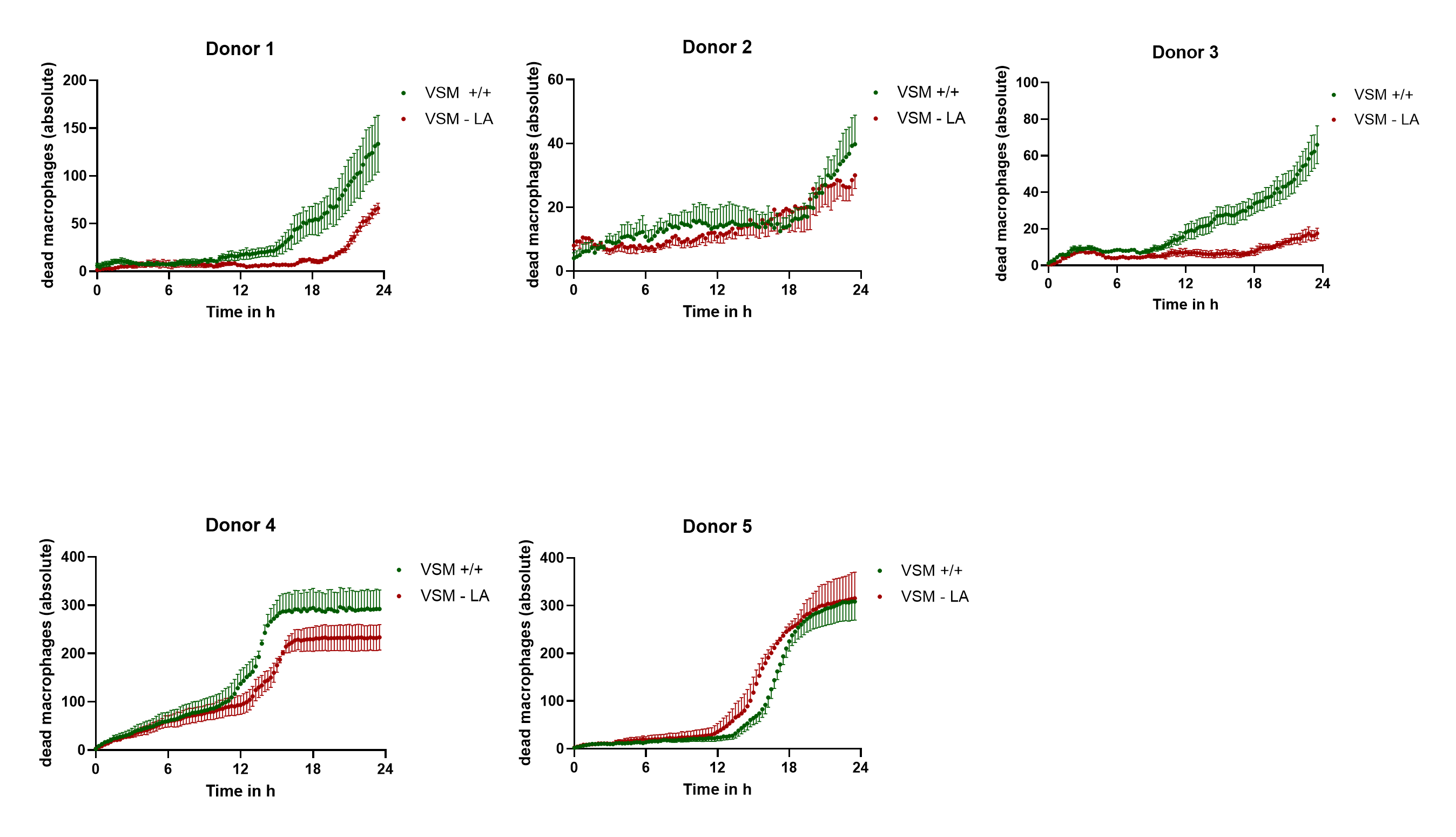

Supplement: Supplementary Figure 1 after revision.tif [file KVIR_A_2451165_SM9631.tif]

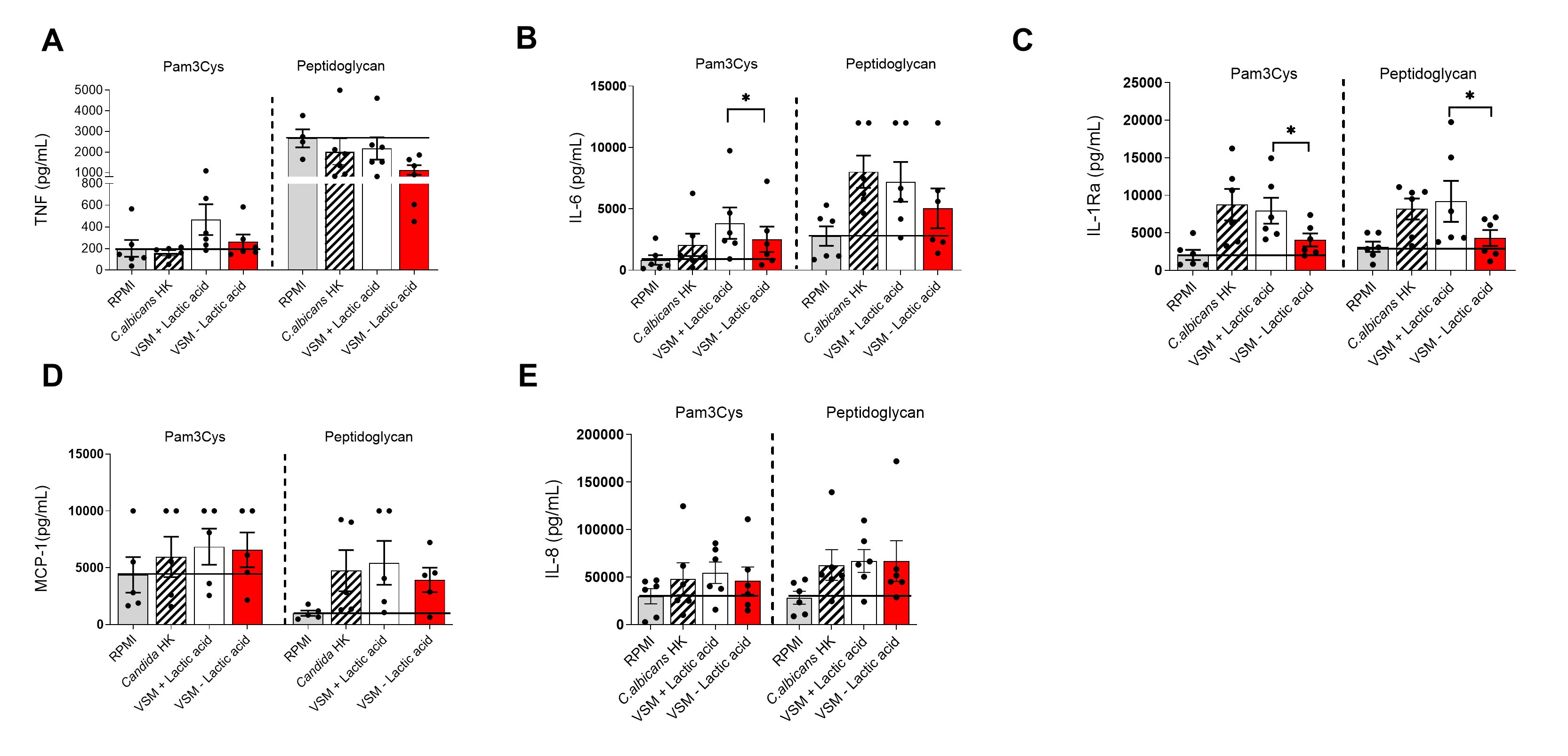

Supplement: Supplementary Figure 2 after revision.tif [file KVIR_A_2451165_SM9630.tif]
